# Changes in Computer-Analyzed Facial Expressions with Age

**DOI:** 10.3390/s21144858

**Published:** 2021-07-16

**Authors:** Hyunwoong Ko, Kisun Kim, Minju Bae, Myo-Geong Seo, Gieun Nam, Seho Park, Soowon Park, Jungjoon Ihm, Jun-Young Lee

**Affiliations:** 1 Interdisciplinary Program in Cognitive Science, Seoul National University, Seoul 08826, Korea; powerzines@snu.ac.kr (H.K.); minju1222@snu.ac.kr (M.B.); ijj127@snu.ac.kr (J.I.); 2 Department of Psychiatry, SMG-SNU Boramae Medical Center, Seoul National University College of Medicine, Seoul 03080, Korea; imkisun@snu.ac.kr (K.K.); coieyu102@cau.ac.kr (M.-G.S.); genam1006@cau.ac.kr (G.N.); 3 Dental Research Institute, School of Dentistry, Seoul National University, Seoul 08826, Korea; 4 Behavioral Neuroscience Program, School of Medicine, Boston University, Boston, MA 02101, USA; sehopark@bu.edu; 5 Division of Teacher Education, College of Liberal Arts and Interdisciplinary Studies, Kyonggi University, Suwon 16200, Korea; swpark1@kyonggi.ac.kr

**Keywords:** facial action unit, facial aging, facial expression, posed emotion

## Abstract

Facial expressions are well known to change with age, but the quantitative properties of facial aging remain unclear. In the present study, we investigated the differences in the intensity of facial expressions between older (*n* = 56) and younger adults (*n* = 113). In laboratory experiments, the posed facial expressions of the participants were obtained based on six basic emotions and neutral facial expression stimuli, and the intensities of their faces were analyzed using a computer vision tool, OpenFace software. Our results showed that the older adults expressed strong expressions for some negative emotions and neutral faces. Furthermore, when making facial expressions, older adults used more face muscles than younger adults across the emotions. These results may help to understand the characteristics of facial expressions in aging and can provide empirical evidence for other fields regarding facial recognition.

## 1. Introduction

Expression and recognition of emotions through facial expressions are fundamental functions of basic communication. Facial expressions are critical for communicating with one’s surroundings in terms of their role to convey the primary meaning of social information [1,2]. People can communicate and convey their emotions in diverse manners; however, facial expressions can be used in the most flexible way [3]. Investigating how facial movements are controlled and how people recognize others’ facial expressions, therefore, is an essential way to understand the nature of human beings as social beings and can also facilitate emotional functioning.

It has been well established that emotional expression and recognition skills through facial expressions change with age [4,5]. A previous study showed older and young people a variety of facial expressions and confirmed how they recognized them [6]. Young and old people were both aware of expressions of positive emotion, while older people were less aware of negative facial expressions. In addition, the performance of the older group declined in sadness facial expression recognition but improvement in disgust facial expression recognition [7,8,9]. The older people were also more inclined to think that they felt happy when they were shown smiles [10]. A recent meta-analysis demonstrated that older adults showed lower performance on emotional face identification than a younger group of adults [11].

Owing to physical aging, sarcopenia, such as atrophy of facial skeleton, malposition of fatty muscles, and loss of soft tissue happen most commonly in the areas of the maxilla, mandible, and anterior nasal spine [12]. A previous study showed that human facial aging demonstrated a common pattern of morphological, chronological, and dermatological changes in various biomedical studies [13]. In an aspect of neuromuscular mechanism, voluntary facial expressions (i.e., posed facial expressions) using the lower part of the face are prominently controlled by the left hemisphere and vice versa [14,15,16]. Specifically, aging of the orofacial motor cortex, which involves involuntary facial expressions, can cause a decline in cognitive control for the lower part of the face [17,18]. While facial aging is natural and inevitable for most people, multiple studies have suggested there are several markers of facial expression and recognition in neuropathological changes including epilepsy [19], Parkinson’s disease [20], Alzheimer’s disease [21], and other neurocognitive disorders [22]. Despite this, identifying the quantitative characteristics of facial aging is still limited.

The posed facial expression, which is commonly exhibited on portrayal of other’s facial expression, has distinct characteristics compared to spontaneous facial expression in aspects of neuromotor system and display rules. Whereas posed facial expression is generated cognitively within the pyramidal system, spontaneous facial expression exhibits independent motor control and is driven by extrapyramidal system [15,23]. The movements inherent to posed facial expression tend to display intended emotions in the context of social interactions (i.e., display rules), while spontaneous facial expression correspond to a primary emotional system [15,24]. Although, several studies have pointed out the limitations of the characteristic of the posed facial expression for its artificiality by actor’s and variability by experimental conditions [25,26,27], research leveraging posed facial expression has clear advantages. For interpretability, posed facial expression is less ambiguous than spontaneous facial expression [28] and is also universal across the basic emotion [29]. Such universality has also been identified in recent study for East Asian population [27]. Since cumulative literatures have studied the pose facial expression [30], posed facial expression is may expected to be a valid indicator for investigating aging.

Quantitative measurements of facial expressions and their analyses has been an active research topic in behavioral science. Among several studies, a facial action coding system (FACS) [31,32] is the most widely used in this area. A series of facial muscle movements that represent facial expressions, termed as action units (AUs), can help a facial recognition-based analysis to be more standardized [33]. Since AUs were originally developed from basic emotion theory and manually rated by highly trained coders, the FACS-based AUs have had limited accessibility for standardization. Recently, automated computer vision and multidiscipline study for facial expression analysis have emerged [34]. These studies enable scaling facial expressions more feasible; facial aging study remains in three-dimensional (3D) morphometric [13,35] or electromyography (EMG) studies [36,37]. In that regard, little is known about quantitative facial aging.

Given that facial expressions are crucial indicators of human health status [38,39], applying machine learning algorithm techniques to facial expressions, such as computer-aided diagnosis (CAD) in the biomedical signal [40], and the medical imaging field [41], can contribute to digital health. This technique is often used in facial paralysis [42,43], face transplant [44], pain detection through facial expression [45], and neurologic studies such as those involving autism [46], Turner syndrome [47], and Parkinson’s disease [48]. Since language production and discourse decrease with aging [49], identifying the characteristics of facial expressions in the older adults is a promising and challenging research area in gerontology, which can diagnose disease regardless of patient communication skills. Moreover, the uniqueness of facial expressions has led to consistent studies in the area of personal identification for health records [50], to improve performances on CAD and identification using facial expressions, to develop the algorithm, and to provide interpretable results for facial expressions with aging. Although there has been much work on automatic facial expression recognition in computer vision research, the algorithms have been experimentally validated primarily on younger faces. For facial expressions to be better used as digital markers related to aging, finding quantitative differences in facial changes with aging should be studied.

The aim of this study was to identify the characteristics of facial expressions based on the basic emotion theory and to compare the differences in facial expressions between younger and older adults for each basic emotion and AU, respectively. Additionally, a feature-selection approach was used to identify multivariate patterns of the changes in facial expressions related to aging. Finally, the predictive ability for selected AUs was evaluated.

## 2. Materials and Methods

### 2.1. Ethics Statement

This study was approved by the Institutional Review Board of the SMG-SNU Boramae Medical Center (IRB No. 30-2017-63), and all participants submitted written consent for participating in the study.

### 2.2. Participants

A total of 61 older adults and 115 younger adults were recruited for this study. The older adults were between 62 and 84 years old and recruited from the Alzheimer’s disease research center of the SMG-SNU Boramae hospital. Healthy young participants were recruited from the university student participant pool and aged between 18 to 39. None of them had a history of psychiatric disorder. Major medical diseases, severe head injury, and visual impairment were excluded in all groups. Especially, all the older adults were free from the diagnosis criteria of Alzheimer’s disease and depressive spectrum disorder with DSM-IV [51]. All medical judgements were determined by a board-certified psychiatrist (J.-Y.L.).

To screen the potential emotion related problems such as depression, anxiety, and alexithymia, participants were asked to answer self-reported measures: Beck Depression Inventory (BDI), Beck Anxiety Inventory (BAI), and Toronto Alexithymia Scale (TAS). The Korean version of BDI involves 21 questions to evaluate the severity of depression, with scores ranging from 0 to 63 [52,53]. A higher score indicates severe depressive symptoms, and the cutoff score is 18 in the Korean version [54]. The Korean version of BAI utilizes 21 questions to measure the severity of anxiety, with scores ranging from 0 to 63 [55]. A higher BAI score indicates severe anxiety symptoms with a cutoff score of 19 [56]. A twenty-item TAS was developed and validated to measure the severity of alexithymia. A score ranging from 20 to 100 [57,58], with a cutoff score at 61 was used for the Korean version [59]. The TAS is made up of three subscales: Difficulty identifying feeling, difficulty describing feeling, and externally oriented thinking. Neither group had an abnormal level of emotional problems (Table 1).

Since data for five older adults and two younger adults failed to pass the quality check, 169 of 176 participants were included in the analysis. Table 1 summarizes the demographic and clinical characteristics of the participants. Significant differences were found in age, education, left-handed, BDI score, and TAS score. Except for age, these variables were adjusted in further analyses.

### 2.3. Procedures

**Figure 1:**
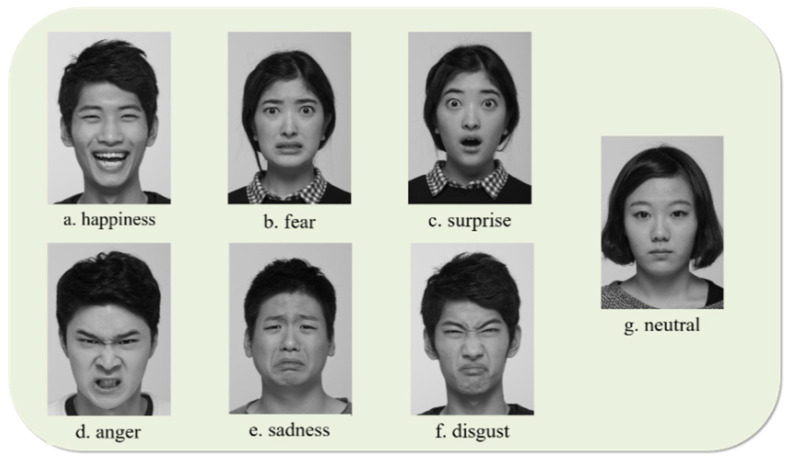
The facial stimuli representing the six basic emotions and the neutral emotion, adapted from [60].

A series of photos containing six basic emotions and a neutral facial expression were presented to participants, which consisted of seven stimuli and had been selected by researchers from a photography dataset used in a previous study [50]. Instructions were given in both verbal and visual form, and the participants were asked to answer verbally for stimuli. Then, participants performed posed facial expressions for the given list of six basic emotions and the neutral emotion. For example, for happy facial expression, a photograph of a person with a happy face was presented; participants were asked to identify the emotion conveyed; and “make a happy face for 15 s towards the camera” to be video recorded. The facial stimuli were given once participants were fully aware of the instruction of the study. Examples of stimuli are shown in Figure 1. Each facial stimulus was presented for a maximum of 7 s; the researcher moved on to the next stimulus when the participant made a verbal response. Facial expressions were acquired for a total of 105 s for each emotion.

### 2.4. Data Acquisition

The participants’ video recordings of posed facial expressions were administered with a Canon EOS 70D DSLR Camera with a 50 mm prime lens, 720 p resolution, and 60 fps frame rate. The camera was positioned on a fixed stand approximately 120–140 cm above the floor to correctly capture the entire face of the participants. The posed facial expressions were recorded for 15 s after a clear instruction to imitate a previously recognized emotional face.

For each frame of the recorded videos, the presence and intensity were estimated using OpenFace 2.0, an open-source toolkit for facial behavior analysis, which consists of four pipelines: (1) facial landmark detection and tracking, (2) head pose estimation, (3) eye gaze estimation, and (4) facial expression recognition [34]. For analyzing facial expressions, OpenFace 2.0 recognizes facial expressions by detecting AU intensity and presence according to FACS [31]. Without using all the AUs listed in FACS, OpenFace 2.0 offers a subset of 18 AUs by cross-dataset learning, specifically, 01, 02, 04, 05, 06, 07, 09, 10, 12, 14, 15, 17, 20, 23, 25, 26, 28, and 45. The occurrences and intensities in AUs are estimated by using machine learning algorithms. The methods for AU estimation and analysis are described in more detail elsewhere [61]. In the present study, AU intensities were used to derive measures of individual emotional facial expression and six basic emotions were created according to emotional FACS (EMFACS) [62]. The EMFACS were based on the FACS that have been proven to have significant reliability for the assessment of human facial movements [63,64]. The highest intensity for each AU was calculated as the maximum score across all the video frames, which is validated in prior work [65]. Examples of each AU and emotion are shown in Table 2.

### 2.5. Statistical Analysis

Descriptive statistics for demographic variables were calculated as mean scores and standard deviations. The difference in AU was compared, applying for multiple comparisons (followed by Bonferroni correction). Chi-squared tests were used to compare categorical outcomes such as sex and usage of botulinum toxin (botox). The correlation between age and the AU intensity was investigated. To explain multivariate profiles with respect to input features that were accurately distinguished from the older group, the adaptive least absolute shrinkage and selection operator (LASSO) ML algorithm were applied to the dataset [66]. The adaptive LASSO, which is a regularized regression method with L1-norm penalty [67] is a popular technique for simultaneous estimation and consistent variable selection [66]. It is a powerful model that performs regularization and feature selection, and it can provide model interpretability by excluding irrelevant features that are not related to the class from the model. L1 regularization, which penalizes elements of redundant complexity, focuses on the most significant features, and thus prevents overfitting of the data and is supported by well-grounded theoretical analysis [68]. The regression coefficients of unimportant variables shrank to 0 upon implementing the adaptive LASSO. In that regard, the adaptive LASSO algorithm provided interpretable results related to the older adults. Due to its high accessibility and low computational complexity as compared with other feature selection models, recently, this approach has been highly recommended in behavioral science [69].

In order to avoid the overfitting issue and to evaluate the generalizability of the results from the ML algorithms, 10-fold cross-validation was applied during the variable selection process [70]. First, the data were randomly split into a training set (66.7% of the data) and a test set (33.4% of the data). All the ML models were fitted using the training set, and classifications were separately made on the test and training datasets. The optimal parameter, lambda, was determined across 1000 iterations of 10-fold cross-validation to minimize the deviance of the model. Then, predictions were made on the test set based on the ML models trained in the training set. All reported *p* values have been adjusted for multiple comparison analyses.

## 3. Results

### 3.1. The Differences in Facial Expression between the Older Adults and Younger Adtuls

**Figure 2:**
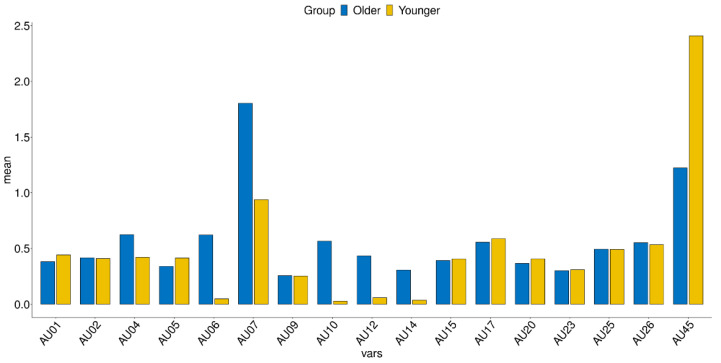
Prevalence of AU values by groups for neutral face. AU, action unit.

**Figure 3:**
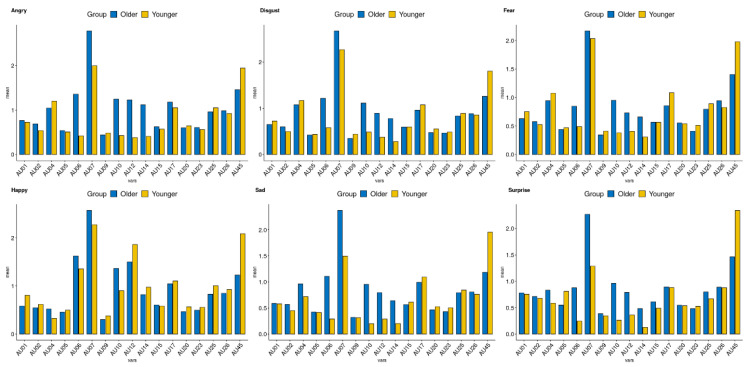
Prevalence of emotional AU values by groups for emotional face. AU, action unit.

Figure 2 and Figure 3 demonstrate the AU values of the older and younger adults for the neutral and emotional face. The results applied for multiple comparisons are presented in Table 3. In AU 06, 07, 12, and 14, older adults showed higher intensity compared to younger adults. For AU 45, older adults showed lower intensity than younger adults.

**Figure 4:**
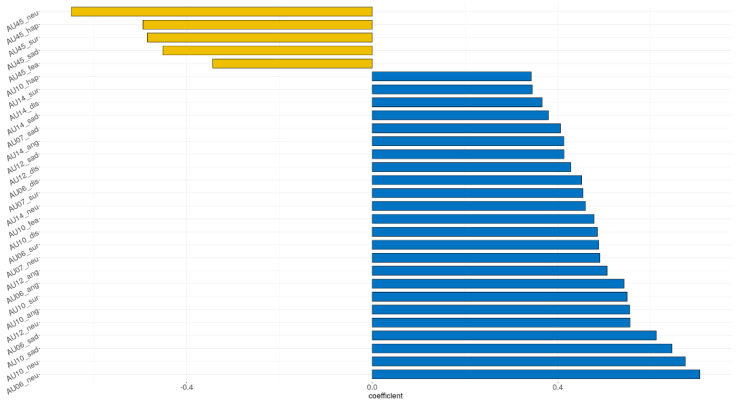
Correlation plot for age and AUs. AU, action unit; ang, angry; dis, disgust; fea, fear; hap, happy; neu, neutral; sur, surprise. *p*-values were adjusted for multiple comparisons.

To explore the relationship between age and each AU, a correlation analysis was conducted. The patterns of the results were similar to differences in group comparisons (Figure 4). For AU 06, 07, 12, 10, and 14, positive correlations between AU and age were found, while negative correlation were found in AU 45 across the emotions.

### 3.2. Feature Selection for Predicting Age

**Figure 5:**
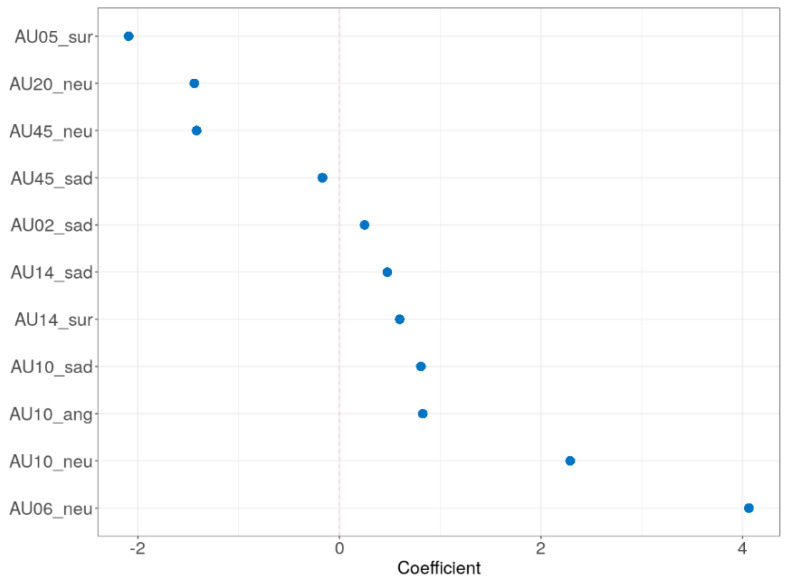
The adaptive LASSO results. AU, action unit; ang, angry; neu, neutral; sur, surprise.

The adaptive LASSO model was implemented to identify significant features for distinguishing the older group among the input variables. Demographics (education, sex, left-handed, and botox), self-reported measure (TAS and BDI), and all AUs were assessed for their ability to classify the older adults. Figure 5 shows the multivariate profiles for distinguishing the older adults from the participants in the current study. Demographics and self-reported measure were not significant in the adaptive LASSO result. Among the total 119 AUs, only 11 AUs remain significant: AU 10 in angry; AU 02, 10, 14, and 45 in sad; AU 05 and 14 in surprise; AU 06, 10, 20, and 45 in neutral, respectively. The receiver operating characteristic (ROC) demonstrated an AUC of 0.924 for the adaptive LASSO model.

## 4. Discussion and Conclusions

The purpose of the present study was to investigate the differences in facial expressions of older and younger adults and to examine how facial muscles contributed to aging through AUs for six basic emotion and neutral facial expression. Throughout the emotions and AUs, the older adults appeared to exhibit greater intensity in facial expression than the younger adults. In some area, the older adults showed lower facial intensity than the younger adults.

### 4.1. Degenerative Changes in Facial Expression Differences with Age

The main findings show that the older adults have higher AU values than young people for neutral and negative emotion (i.e., angry and sad). An increasing amount of the literatures has demonstrated that aging is associated with dramatic reductions in muscle strength (i.e., dynapenia) and motor control [71,72,73]. With advancing age, decreased neuromuscular changes may result in deficits in voluntary activation for facial activities [73,74]. In that regard, the facial expressions of older adults can naturally differ from those of younger adults [75].

Given that the cortex, spinal cord, and neuromuscular junction are functionally correlated, and they influence voluntary activation of muscle fibers [76], voluntary facial expressions can be addressed by neurological evidence [77]. For older adults to make facial expressions as intended, therefore, it is necessary to utilize their brain in the top-down processing format to ensure that the commands from the brain are correctly delivered to the facial muscles. In addition to facial aging due to sarcopenia, this suggests that changes in the motor cortex with aging can cause changes in facial expressions in the older adults [78,79].

Regarding the expression of strong negative emotions in the older adults representing our results, age differences are reported between the older and the young adults when they discriminate negative emotion. A previous study demonstrated that older adults had more difficulty distinguishing low intensity negative emotions [80]. They may tend to make facial expressions excessively because the older adults themselves may not be able to identify low intensity negative emotions.

Previous studies well support the differences in AUs intensity between the two groups. On upper facial expression, namely AU 06 and 07, the older adults can show greater intensity than the younger adults. Increased activity in orbicularis oculi muscle [81], deeply set of eye [82], and changes in eyelid due to poor visual acuity [83] may have affected the changes in upper facial expression. For lower facial expression, AU 10, 12, 14, the strength of the face may have been further tapped due to the highlighted facial contour caused by loss of subcutaneous fill around the nose and mouth in the older adults [84]. In AU 45, the older adults rather showed reduced AUs than the younger people. Elevated duration of eye blink may explain this reason. Duration of the eye-blinking decreases with aging, apparently reflecting decreased intensities in AU 45 [85], since the deterioration of the orbicularis oculi muscle can affect the complete eye closure rate [86].

As for the adaptive LASSO, the result was shown to be similar to the comparisons between two groups, expect for the AU 02, 05, and 20. The increase in AU 02 in sad condition, as previously mentioned, may have resulted in increased activity in the eyebrow and strong representation of negative representations [80,81]. For the AU 05 in surprise condition, the reduction of muscles may also involve in eye activity have affected the weaker construction of surprise facial expressions [85,86]. For the AU 20, aging may lead to the relaxation of the lip stretcher owing to decreased muscle around the mouth [17,87].

### 4.2. Limitations and Future Direction

There are several limitations in the current study. First, we employed only posed emotions. Given that the mechanisms of the posed emotions and the spontaneous facial expressions differ [88], further studies are needed to compare the difference between two distinct facial expressions. Secondly, we did not employ physiological assessment. The OpenFace software, unlike EMG, could not measure sensitive intensities in facial muscles at a physiological level. However, since the OpenFace library is based on FACS and provides reliable results along with recent technological advances, measurement errors are not likely to be a problem. In addition, recent study on the difference between computer vision and EMG has demonstrated only a few differences among the two techniques with respect to accessing overt facial expressions, and that computer vision showed better performance as compared with human [89]. Thirdly, age group is less continuous. Thus, future studies should be designed for providing normative data for facial aging with respect to demographics, such as age and sex. Lastly, the presence of the imbalanced class between the younger group and older group can be a potential limitation of the current study. This issue may not be critical, if the ratios between two classes are not too different. An experimental study showed that low class imbalance ratios do not cause significant performance loss [90], where the class ratio of 40:60, which is similar to our study (Table 2), seemed to converge to nearly zero with respect to performance loss. Another study used metabolomics data and showed that a false positive ratio even decreases as the class-imbalanced ratio rises, due to the prevention of over selection in identifying biomarker features with the LASSO algorithm [91]. Despite these studies, our findings should be interpreted with caution.

With the above limitations, our study has the following strengths. Our findings regarding posed emotions, which require conscious effort of facial muscles, can be used as an evidence to censor individuals who deliberately deceive others, especially for lie detection [92]. In situations where biophysiological assessment is limited, computer vision-based face recognition tools would be beneficial. In a clinical setting, our findings can be used for detecting frailty and other senile changes in muscle. For computer vision-based facial recognition, our findings may also provide researchers with empirical evidence for the characteristics of a human aging face, which would help develop the service and/or product for recognizing the faces of older adults. Notably, so far, there has been little attempt for facial expression recognizing study that compares the characteristics between the younger and the older. Our findings can provide interpretable evidence and explainable features for aging faces. This could provide an important basis for CAD studies for older people in the future.

### 4.3. Conclusions

Taken together, the present study is the first to investigate the differences in posed facial expressions between older adults and younger adults using a computer analysis method. Our findings provide evidence for implications in facial expression intensity based on FACS-AU-derived emotional faces. The older adults expressed more intense expressions in neutral and negative emotions than younger adults and tended to use more muscles when they were making facial expressions. In some part of the facial expression, the older adults showed weaker intensity than the younger adults. Our findings may suggest that changes in the muscles around the eyes and mouth due to aging can be indicators of the characteristics for identifying the aging face. The results of this study were obtained quantitatively from a normal population, which has several strengths as compared with previous studies of facial expression based on EMG, 3D morphometry, or subjective rating. They can be used as a basic methodology for analyzing and for identification of the characteristics of facial aging. We hope that the various features of the posed emotions of the older adults in this study can be a significant contribution to other scientific fields with respect to facial expressions, such as criminological research using lie detection, behavioral medicine, and computer vision research based on facial recognition. Future studies are needed for investigating other attributes in facial expressions regarding dynamic emotions, natural environments, and diverse groups.

## Figures and Tables

**Table 1:** Demographic characteristics across the groups.

		Younger Adults (*n* = 113)	Older Adults (*n* = 56)
		Mean ± SD	Mean ± SD
Age		**21.9** **± 2.9** **1**	**72.** **2** **± 4.** **72**
Education		**14.5** **± 1.10**	**9.8** **± 4.47**
Sex, *n* (%)			
	Male	57 (50.4)	27 (48.2)
	Female	56 (49.6)	29 (51.8)
Usage of botox, *n* (%)		2 (1.8)	1 (1.7)
Left-handed, *n* (%)		8 (7.1)	1 (1.7)
BDI		**10.** **7** **±** **6.88**	**14.** **4** **± 11.0** **4**
BAI		25.1 4.28	25.3 ± 6.22
TAS		**45.** **3** **± 10** **.52**	**50.3** **± 8.9** **5**

Note. Botox, botulinum toxin; BDI, Beck Depression Inventory; BAI, Beck Anxiety Inventory; TAS, Toronto Alexithymia Scale; SD, standard deviation; BOLD indicates statistically significant differences.

**Table 2:** Action unit descriptions and combination of each emotion.

No.	FACS Name	Facial Muscle (Location)
1	Inner brow raiser	Frontalis, pars medialis (U)
2	Outer brow raiser	Frontalis, pars lateralis (U)
4	Brow lowering	Depressor glabellae, depressor supercilli, currugator (U)
5	Upper lid raiser	Levator palpebrae superioris (U)
6	Cheek raiser	Orbicularis oculi, pars orbitalis (U)
7	Lid tightener	Orbicularis oculi, pars palpebralis (U)
9	Nose wrinkle	Levator labii superioris alaquae nasi (L)
10	Upper lip raiser	Levator labii superioris, caput infraorbitalis (L)
11	Nasolabial deepener	Zygomatic minor (L)
12	Lip corner puller	Zygomatic major (L)
14	Dimpler	Buccinator (L)
15	Lip corner depressor	Depressor anguli oris (triangularis) (L)
17	Chin raiser	Mentalis (L)
20	Lip stretcher	Risorius (L)
23	Lip tightener	Orbicularis oris (L)
25	Lip parting	Depressor labii, relaxation of mentalis, orbicularis oris (L)
26	Jaw drop	Masetter, temporal and internal pterygoid relaxed (L)
45	Blink	Levator palpebrae superioris, orbicularis oculi (U)
Emotion	AU combination	
Angry	04 + 05 + 07 + 23	
Disgust	09 + 15	
Fear	01 + 02 + 04 + 05 + 20 + 26	
Happy	06 + 12	
Sad	01 + 04 + 15	
Surprise	01 + 02 + 05 + 26	

Note. AU, action unit; FACS, facial action coding system; L, lower face; U, upper face.

**Table 3:** AU comparisons by groups for six basic emotions.

Variables	Younger Adults	Older Adults	Direction	Location	*p*-Value
	Mean ± SD	Mean ± SD			
AU06 (ang)	0.42 ± 0.58	1.36 ± 0.85	Y < O	U	<0.001
AU06 (dis)	0.58 ± 0.54	1.22 ± 0.73	Y < O	U	0.0276
AU06 (neu)	0.05 ± 0.14	0.62 ± 0.45	Y < O	U	<0.001
AU06 (sad)	0.29 ± 0.45	1.11 ± 0.59	Y < O	U	<0.001
AU06 (sur)	0.25 ± 0.50	0.88 ± 0.59	Y < O	U	<0.001
AU07 (neu)	0.94 ± 0.70	1.80 ± 0.83	Y < O	U	<0.001
AU07 (sad)	1.50 ± 0.94	2.37 ± 1.03	Y < O	U	<0.001
AU07 (sur)	1.29 ± 0.94	2.27 ± 0.97	Y < O	U	0.0105
AU10 (ang)	0.43 ± 0.58	1.25 ± 0.61	Y < O	L	<0.001
AU10 (dis)	0.49 ± 0.50	1.11 ± 0.62	Y < O	L	<0.001
AU10 (fea)	0.38 ± 0.49	0.95 ± 0.58	Y < O	L	<0.001
AU10 (neu)	0.03 ± 0.13	0.57 ± 0.47	Y < O	L	<0.001
AU10 (sad)	0.20 ± 0.34	0.95 ± 0.55	Y < O	L	<0.001
AU10 (sur)	0.26 ± 0.46	0.96 ± 0.61	Y < O	L	<0.001
AU12 (ang)	0.38 ± 0.56	1.23 ± 0.83	Y < O	L	<0.001
AU12 (neu)	0.06 ± 0.15	0.43 ± 0.40	Y < O	L	<0.001
AU12 (sad)	0.29 ± 0.43	0.79 ± 0.65	Y < O	L	<0.001
AU14 (ang)	0.41 ± 0.63	1.12 ± 0.81	Y < O	L	0.0255
AU14 (neu)	0.04 ± 0.15	0.31 ± 0.38	Y < O	L	<0.001
AU14 (sad)	0.20 ± 0.41	0.64 ± 0.60	Y < O	L	0.0036
AU45 (hap)	2.09 ± 0.70	1.23 ± 0.63	Y > O	U	0.0029
AU45 (neu)	2.41 ± 0.69	1.22 ± 0.55	Y > O	U	<0.001
AU45 (sad)	1.95 ± 0.75	1.18 ± 0.61	Y > O	U	0.0495
AU45 (sur)	2.34 ± 0.77	1.46 ± 0.73	Y > O	U	0.0022

Note: AU, action unit; BOLD, indicates significant *p*-values; ang, angry; dis, disgust; fea, fear; hap, happy; neu, neutral; sur, surprise; L, lower face; U, upper face. Comparisons were adjusted for covariates. *p*-values were adjusted for multiple comparisons.

## Data Availability

The data presented in this study are available on request from the corresponding author.
